# The Dentist and the Orthodontist

**Published:** 1916-02

**Authors:** Martin Dewey


### CO-OPERATION BETWEEN THE DENTIST AND THE ORTHODONTIST.

BY MARTIN DEWEY, D.D.S.

If the dentists and orthodontists are to render the greatest possible service to the public, there must necessarily be co-operation between the two. The necessity for this co-operation is apparent to every one connected with these two professions, but in times past there has not been the close relation existing between the orthodontist and dentist that there should have been. There have been several reasons for the differences which have existed between the two groups, whose work is very closely related and whose success is often interdependent.

It is the dental practitioner who first observes cases of malocclusion. In fact, the general practitioner who has reached the point of being a "family dentist" will be in a position to first observe malocclusions that develop in young children. If he is desirous of doing the greatest service for patients who may require orthodontia treatment, he should have a consulting acquaintance with some competent orthodontist to whom he can refer his patients at the proper time, so that they may be treated with the least trouble to the patients and obtain the best results in the shortest time conditions will permit. There are, however, some dentists who refuse to follow this plan, and we have even known of men who, when they found malocclusion in the mouths of their patients, would not refer them to an orthodontist for several reasons, the principal reason being that they take a purely mercenary view of the situation. Some have argued, "Why should I send this case to the orthodontist, or why should I send to some one else what I can not do myself?" In other words, the dentist is not capable of correcting the malocclusion, yet he would allow the defect to remain rather than refer it to one who could probably treat it. This question of financial consideration has often been the bone of contention—whether orthodontists should pay commissions to general practitioners. As we do not desire to discuss this phase of the question at this time, we will simply state that the man whose perspective of malocclusions in cases of his patients is from the financial standpoint will not necessarily work for the best interests of his patients.

Other instances where co-operation between orthodontists and dentists is very beneficial to the patient are in those cases in which malocclusions are occurring or may be produced because of prolonged retention or early loss of the deciduous teeth. We have seen a great many cases of malocclusion produced by the early extraction of deciduous teeth, which could have been avoided if the dentist had consulted an orthodontist who was familiar with the manner in which malocclusions develop. We have also seen malocclusions grow from a minor defect to a serious condition simply because improper advice was given by the dentist, such advice often being for the patient to "wait awhile and the case will correct itself." If, therefore, an orthodontist were consulted in cases of malocclusion, in many cases in which his line of work qualifies him to understand the conditions better than are known to the general practitioner a greater amount of good would be accomplished for the patient, and, in fact, for humanity.

We must not blame the general practitioner or the dentist entirely for this lack of co-operation, for we are aware that there are many men practicing orthodontia who do not desire to be called into consultation with the general dentist unless there is a fee involved. Some orthodontists positively refuse to give advice in regard to the treatment of a case unless they can immediately obtain a fee. The orthodontist who takes this narrow view of life is certainly not disposed to benefit the community, or elevate his profession, and in a short time will find that a man with a broader view will gain his practice.

There is also an attitude assumed by orthodontists that they occupy a superior plane to that of the dentist—or, in other words, belong to a selected group—and do not care to communicate, advise, or consult with a dentist in regard to any case. We have also known of strained relations to occur between orthodontist and dentist after the dentist has referred a case to an orthodontist. The orthodontist often assumes entire charge of a patient, and does not to any extent consult the family dentist. We do not consider it advisable for the family dentist to attempt to advise the orthodontist how a malocclusion should be treated, but we feel that the orthodontist should show some consideration to the general practitioner in regard to fillings, bridge work, etc. We have known cases where general practitioners sent patients to a certain orthodontist, and this orthodontist referred the patients to another practitioner because the second practitioner was the orthodontist's personal friend. This is very unfortunate, for, if there is to be co-operation between the orthodontists and dentists, the patient referred to the orthodontist should certainly be sent back to the man who referred him and not to someone else. We often find that an orthodontist objects to referring a patient back to the dentist on the grounds that the dentist in question does not do the character of dental work deemed necessary by the orthodontist after the malocclusion is treated. We are aware that many a case of malocclusion will be either a success or a failure, depending on the kind of dental work subsequently done, and there should be co-operation between the orthodontist and dentist in restorations. While the orthodontist is not supposed to know all about dental restoration, he should know something about the forces of occlusion and what must be done in order to treat the case successfully. The dentist who, therefore, fails to avail himself, in making artificial restoration and in building crowns, bridges, or inlays, of the knowledge which the orthodontist possesses regarding the laws of occlusion, will undoubtedly not render the greatest possible benefit to his patients. The dentist should be willing to observe the procedure by which the orthodontist desires restoration to be made so as to make the occlusion of the teeth self-retentive, and not advance the argument that because the man is an orthodontist he knows nothing about dentistry. The orthodontist is also in the wrong at times by failing to realize that the dentist is not familiar with cases of occlusion or the manner in which such cases should be treated. Then again, very often the family history of a case and idiosyncrasies of the patient, which are known to the dentist, would be of greater assistance to the orthodontist if he knew them.

In conclusion we will say that the most intimate relation concerning the consultation between the general dental practitioner and the orthodontist, the better it will be for the dental profession and the general public.—*International Journal of Orthodontia.*

